# Nurses' Safety in Caring for Tuberculosis Patients at a Teaching Hospital in South West Nigeria

**DOI:** 10.1155/2020/3402527

**Published:** 2020-06-16

**Authors:** Risikat Idowu Fadare, Oluwaseyi Abiodun Akpor, Ifeanyi Goodness Ifechukwude, Agbana Richard D, Cecilia Bukola Bello

**Affiliations:** ^1^Department of Nursing Sciences, College of Medicine and Health Sciences, Afe Babalola University, Ado-Ekiti, Nigeria; ^2^Department of Public Health, College of Medicine and Health Sciences, Afe Babalola University, Ado-Ekiti, Nigeria

## Abstract

**Background:**

Tuberculosis remains the leading cause of death due to infectious diseases worldwide ranking above HIV/AIDS, and Nigeria is rated as the 7^th^ worldwide and the 2^nd^ in Africa among the 30 countries highly burdened with tuberculosis worldwide.

**Aim:**

To investigate the challenges encountered by nurses in the care of TB patients in a Federal Teaching Hospital in Nigeria. *Setting*. Ekiti State, Southwest Nigeria.

**Methods:**

A qualitative contextual method was utilized with the sample size determined by data saturation. Data collection was done through an audiotaped, semistructured interview. The study sample consisted of 20 professional nurses working in the medical and paediatric wards of a selected Federal Teaching Hospital in South West, Nigeria. Data was analysed using Tesch's content analysis approach.

**Results:**

The majority of the participants were females within the age group of 31–40 years. Challenges included inadequate availability of personal protective equipment (PPE), lack of isolation wards, delegating the care of tuberculosis patients to young inexperienced nurses, long process in diagnosing patients with tuberculosis, lack of policies protecting the nurses from exposure to tuberculosis, and inadequate training. The major concern was the fear of contracting tuberculosis.

**Conclusion:**

The study suggested that there should be a provision of adequate personal protective equipment; tuberculosis designated wards and provision of periodic training to update the nurses on care of tuberculosis patients. Establishment and execution of hospital policies and practices along with support are equally essential in facilitating a safe workplace for nurses.

## 1. Introduction

Tuberculosis (TB) is a communicable bacterial disease caused by *Mycobacterium tuberculosis (MTB)*. As an airborne disease, TB is transmitted from person to person via droplets from the throat and lungs [1]. Globally, TB is ranked first among the causes of deaths attributable to communicable diseases and the highest killer disease that occurs as a result of a single infectious agent worldwide. [2].

In 2017, about 10 million people manifested symptoms of TB and 1.3 million deaths occurred as a result of the disease [2]. More than 95% of the infected people are from Africa due majorly to poor living standards and high prevalence of HIV/AIDS [2]. A report of the World Health Organization (WHO) stated that, in 2018, Nigeria ranks the 7^th^ out of the 30 high-burden TB countries in the world, with TB prevalence being estimated at 330/100,000 individuals and the number of TB-related deaths at 70/100,000 persons [3]. Another report [4] projected that an incidence of 600,000 cases of all forms of TB occurred in Nigeria with only 91,354 cases being placed on treatment [4, 5].

However, much has been achieved within the framework of the Millennium Development Goals (MDGs) as the TB death rate declined by 45% between 1990 and 2012. This could be attributed partly to the implementation of the comprehensive “Stop TB Strategy” promoted by the WHO [6, 7]. The Directly Observed Treatment Short-course (DOTS) has saved over 22 million lives, owing to the strategy. DOTS is a method in which the health care providers and significant others watch the patients swallow their medications. The method serves as a preferred means of monitoring patients' adherence. There have been reports of some levels of effectiveness through these observations despite its own challenges, some of which are high cost of drugs, staff requirement, and potential for stigmatization [8–10].

Prompt treatment and adherence to the medication regimen guarantee the cure of all forms of TB cases. The treatment duration should be at least six months and the medications must be taken once daily; however, if the patient's sputum remains positive for active TB after two months of management, the treatment may be extended to nine months. According to the WHO [2], TB treatment requires anti-TB medications, provision of information, and supervision and support by the health care providers to the patients [2]. Incorrect use of TB drugs or untimely interruption of treatment can cause drug resistance, thereby increasing the chances of relapse [11]. Moreover, the usage of substandard drugs can lead to MDR-TB [12]. In addition, the use of Directly Observed Therapy enables the nurses or health care providers to observe and ensure that patients take their drugs regularly so as to enhance treatment adherence resulting in successful treatment. This also guards against the development of MDR-TB [13].

Nurses play crucial roles in the management, care, and support of TB patients during the course of their treatment; nevertheless, there are various barriers that can hinder the efficient performance of these roles [14]. According to Makhado et al. [14], nurses' challenges become more obvious due to their direct and close involvement in the care of patients in environments that are usually characterized by shortages in both human and material resources. However, nurses' experiences with respect to this situation as well as the patients' perspective with regard to their treatment processes are rarely investigated.

Therefore, this study investigated the challenges faced by nurses in the care of TB patients in a Federal Teaching Hospital in Southwest Nigeria. It is hoped that findings from this study will provide the basis for the appraisal of hospital policies especially in relation to occupational health and safety of staff and other patients. Also, it is thought that well-implemented recommendations based on the study findings may contribute to a safer work environment and practices which may eventually result in improved quality patient care and nurses' job satisfaction.

### 1.1. Theoretical Underpinning

The health promotion model provided the theoretical underpinning for the study [15]. The health promotion model holds that every individual's action is a product of that individual's distinctive characteristics and experiences. Pender's model focuses on three areas, that is, individual's characteristics and experiences, behaviour specific cognitions and affect, and the behavioural outcomes, and four assumptions [1].

The first part of the model talks about the individual's characteristics and experiences. It includes the individual's past behaviour, personal genetic factors, personal psychological factors, and personal sociocultural factors. The second part talks about behaviour cognition and affect while the third part talks about behavioural outcomes. The health promotion model as used in this study helps to discuss the importance of nurses adhering to universal precaution when caring for TB patients and elicit suggestions about what influences the behaviour of nurses. Perceived benefits of action in relation to this study refer to the nurses' anticipated positive outcomes that will occur when they effectively cater for TB patients while perceived barriers are difficulties that nurses encountered when caring for TB patients. Perceived self-efficacy occurs when nurses are knowledgeable and well equipped to care for TB patients while activity-related affect refers to whether nurses considered their responsibilities in the management of the patients as tasking and stressful or as an enjoyable and fulfilling experience. Conversely, if the experience is seen as tasking or difficult, then it will definitely affect the patient's care. Likewise, interpersonal influence in relation to this study encompasses what motivates the nurses to care for TB patients as well as their perceived risk of being infected or infecting others, while situational influences refer to what directly influences nurses to care for the patients. At this point, the government as well as the hospital management plays a vital role as their contributions can have either a positive or negative influence on the nurses.

## 2. Research Methods and Design

### 2.1. Study Design

The qualitative explorative research design used audiotaped, semistructured interviews to explore the challenges of nurses in the care of TB patients.

### 2.2. Setting

The study was conducted in a Federal Teaching Hospital in Southwest Nigeria. The zone is one of the six geopolitical zones in Nigeria and states including Ekiti State. Culturally, the inhabitants of Southwestern Nigeria are predominantly Yoruba-speaking people who are majorly farmers with few being self-employed and civil servants. Data were generated from one of the two Federal Teaching Hospitals in Ekiti State. The hospital is a 280- bedded tertiary institution consisting of 24 serviceable wards (which include TB units for adults and children) as well as other ancillary units. According to a national survey on TB prevalence in Nigeria, TB prevalence in the Southwest region is 450 per 100,000 while the notification is 40 per 100,000 [16]. The Southwestern region has approximately the same level of TB case notification rate as North Central but a much higher burden of TB prevalence.

### 2.3. Selection and Description of Participants

The target population for this study was nurses working in the hospital. TB patients were admitted in a separate part of the medical wards especially if there are comorbidities such as pleural effusion or due to the severity of the disease or if the disease is detected on admission. Also, TB patients could be admitted if there is extrapulmonary TB. The hospital now practices DOT for TB management. According to the information provided by the hospital record department, a total of 801 patients (447 males and 354 females) were treated for TB in 2019. Criteria for the inclusion in the study were as follows:

- The participant must be a professional nurse
- The participant must be working in any of the selected wards, providing direct care to TB patients
- The participant must be willing to participate in the study.

Purposive sampling method was employed in this study. Before the data collection, the researchers had booked an appointment with the heads of the wards to ascertain the availability of the nurses. All the participants gave their consent to be interviewed and data saturation was used to determine the sample size. Data saturation was achieved when 17 participants had been interviewed. Data collection and analysis were done concomitantly. Within 24 hours, the transcription of recorded information was completed. Data saturation was attained when no new information was forthcoming from the participants, and this was based on all interview items on the challenges of nurses and strategies to overcome them. Thus, 17 interviews were analysed with new categories and 3 interviews were analysed without new categories emerging. Referential adequacy was achieved, partially satisfying the requirement of trustworthiness.

### 2.4. Collection of Data

Prior to data collection, the research details were given to the participants and they gave their consent to participate. The collection of data took place between January and March 2016. The nurses working in the medical and paediatrics wards of the hospital were interviewed, using an interview guide. The interview guide contained some questions regarding the nurses' concern about their safety when caring for TB, awareness of MDR-TB, ways through which nurses might be exposed to TB, ways where nurses support TB patients, available measures and policies safeguarding nurses and other patients, and suggested strategies for overcoming the nurses' challenges. Probes and prompts were used to explore more information from the participants. Prior to the actual interview, pretest interviews were conducted using two individuals who had similar characteristics with the participants but who were not eventually included in the final data collection. The interview with each participant lasted between 30 and 45 minutes. The interview responses were audiotaped and field notes were taken following an informed consent from each interviewee. After the interviews, member checking was done in order to ensure trustworthiness.

### 2.5. Data Analysis and Trustworthiness

The interviews were transcribed verbatim by RIF and IGI and samples of the transcripts were given to OAA for verification and coding. Data were analysed using Tesch's content analysis approach. Themes were generated from the data, and they were first identified by RIF and later confirmed by OAA. To ensure trustworthiness, a casual and friendly environment was ensured to gain the trust and confidence of the participants. Ethics committees of the institution and hospital reviewed and approved the research proposal, and the accuracy of the interview responses was done as the participants checked their contribution before transcription of the audiotapes. The researcher's authority, dense description of data, structural coherence, and dependability audit were also employed as strategies to ensure trustworthiness.

### 2.6. Ethical Consideration

Prior to the commencement of the study, the researcher obtained ethical approval from the Ethics Committee of Afe Babalola University and from the Research and Ethics Committee of the Federal Teaching Hospital. Before starting the interview, participants' rights, details of the study, and the role of the participant were explained to the participants, and informed consent and the permission to use the audio recorder were obtained. For privacy, interviews were conducted in a quiet and private room where only each participant and the researcher were present.

## 3. Results

### 3.1. Sociodemographic Data

As shown in Table 1, a majority (14 of the 20) of the participants were females within the age 31–40 (13 of the 20), 10 of the 20 were in Nursing Officer 1I cadre, and less than half (9 of the 20) of the participants were working in the male medical ward. The majority (16 of the 20) had 1–10 years' experience working with TB patients.

The findings of the study are presented according to the themes and subcategories generated from the data as shown in Table 2.

### 3.2. Theme 1: Challenges of Nurses

#### 3.2.1. Concern about Safety When Working with Tuberculosis Patients

When nurses were asked if they have ever been worried about their safety in their care of TB patients, all of the 20 respondents said they are always concerned about their safety when working with TB patients. Some of their responses were as follows:

  “Yes now, it is very important as a nurse to consider your safety first.”(p2, male, nurse)
  “Yes *o*, our health is of utmost importance.”(p 3, male, nurse)

#### 3.2.2. Years Working with Tuberculosis Patients

With regard to the participants' length of working experience with TB patients, almost half of the participants (9 of 20) said they have been working with TB patients for 5–7 years. Below are two of their responses:

  “Ever since I've been working on this ward so like 7 years.” (p15, male, nurse)
  “I have been working in this unit for 6 years.” (p18, male, nurse)

#### 3.2.3. Knowledge of MDR-TB

When the participants were asked if they have heard of MDR-TB, the majority (16 of the 20 participants) said yes, they have heard of MDR-TB. Some of their responses were as follows:

  “Yes, I have heard of it, it's the one that involves use plenty drugs for.” (p3, female, nurse)
  “Yes, it is very dangerous.” (p2, male, nurse)
  “Yes, I know of it.” (p1, male, nurse)

#### 3.2.4. Perception of MDR-TB as a Challenge at Their Workplace

When the nurses were asked if MDR-TB could be a problem at their workplace, 6 of the 16 participants said yes, MDR-TB is a problem at their workplace.

Some of their responses were as follows:

  “Yes, it is a problem because there is no treatment centre; here you can only take in patients in the continuation phase because people with MDR-TB are cared for in two phases, the admission phase, where they stay in the wards for 6 months and the continuation phase. It is a problem because people with MDR-TB have to go to Abeokuta or LUTH or UCH, Ibadan.” (p8, female, nurse)
  “Yes, because it is more difficult to manage.” (p9, female, nurse)
  “Yes, it is actually a problem.” (p19, female, nurse)

#### 3.2.5. Ways through Which Nurses Might Be Exposed to Tuberculosis at Work

Half of the participants (10 of the 20) said when nurses do not follow necessary precautions like using face masks when caring for TB patients, they can be exposed to TB. Some of their responses were as follows:

  “Practicing good infection control. You must practice good hand washing and nurse the patient in an open space. Also, you must wear your face mask and give the patient face masks too. In essence, the nurse must strictly avoid negligence.” (p1, male, nurse)
  “A nurse can be exposed when there is inadequate ventilation and lack of materials for universal precautions like face masks.” (p6, male, nurse)
  “A nurse can be exposed when the nurse does not use face masks to nurse them and when she attends to a patient with undiagnosed tuberculosis.” (p10, female, nurse)

#### 3.2.6. Activities That Constitute TB Exposure

When participants were asked if there are any activities in particular that can lead to TB exposure, about one-third of the participants (7 of the 20) said that the normal activities on the ward can lead to exposure. Some of their responses were as follows:

  “When you're carrying out normal activities like serving of bedpan and others and you do not practice proper handwashing.” (p3, female, nurse)
  “Normal or routine activities being carried out on the wards in the care of patients especially if the nurse is poorly nourished” (p7, female, nurse)

#### 3.2.7. Knowledge of Whom the Nurses Fear Might Get Infected If They Contract TB

When the participants were asked of whom the nurses feared might get infected if they contract TB, 13 of the 20 expressed that they feared to infect the members of their immediate families and other patients if they contracted TB. Some of their responses were as follows:

  “My immediate family now because after work I would go back to them” (p1, male, nurse)
  “My relatives, I'm scared of infecting my people” (p5, female, nurse)
  “Other patients and my relatives” (p9, female, nurse)

#### 3.2.8. Perceived Challenges of the Nurses

On the challenges faced when caring for tuberculosis patients, diverse answers were given but almost half (7 of the 20) of the participants talked about inadequate personal protective equipment (PPE) to care for the patients, while 8 expressed concern for the effects of nurses' fear on the care of the patients. Some of their responses include the following:

  “This is a communicable disease we are talking about, and in communicable diseases, we have principles, the treatment, the kit, and the procurement. You must protect yourself when caring for tuberculosis patients, and if there are no adequate materials, we cannot care for them adequately.” (p11, male, nurse)
  “There are no isolation wards and no adequate materials for precaution.” (p14, female, nurse)
  “There are no adequate equipment for precaution and also the area of stigmatization among nurses. Once they know that a patient has tuberculosis, they would not want to move closer to the person and they would be avoiding the patient.” (p16, female, nurse) “If a superior officer is assigned to the patient, they would reassign the case to a junior nurse.” (p17, female, nurse)
  “In occupational health, it's your safety first, you have to protect yourself so that you do not become a victim or hazard in the course of helping others so you would not want to go near the patient anyhow, and when you go, you would not stay long with the patient.” (p6, male, nurse)

### 3.3. Theme 2: Roles of Nurses in Supporting Treatment

#### 3.3.1. Giving Health Talks to Patients Concerning the Effect of Noncompliance with Medication Regimen

The nurses were asked if they give health talks to patients concerning the effects of noncompliance with their medication regimen and all the participants (20 of 20) said they do.

Two of their responses were as follows:

  “Yes, you must let them know about the disease condition, its mode of transmission, preventive measures, and the importance of adherence. You must tell about that if they do not adhere to the treatment regimen, it could lead to complications and death. You must be very sincere with them.” (p8, female, nurse)
  “Yes, it is very important. Before drugs are given, the patients are educated on how to comply with the medication because if they miss a dose, the others might be ineffective, so they are enlightened on the effect of noncompliance.” (p4, female, nurse)

#### 3.3.2. Follow-Up and Tracing of Patients That Fail to Come for Medications

The nurses were asked if they ask their patients why they fail to come to the hospital for health talks and all the participants (all of the 20) said they always ask the patient about the reasons for failure to come to the hospital for their medication. Some of their responses were as follows:

  “Yes, we try to reach their mind to see what's happening so that we can know how we can help.”(p2,male, nurse)
  “Yes, if they do not come sometimes, they are called on the phone and traced.” (p8, female, nurse)
  “Yes, it is very important.” (p1,male, nurse)

#### 3.3.3. Health Education on Ways of Preventing the Spread of TB in the Homes and Communities

The nurses were asked if they teach the patient ways of preventing the spread of TB in their homes and communities and all the participants (all of the 20) said they do. Some of their responses were as follows:

  “Yes, immediately they come in for the test; they are taught ways of preventing the spread of tuberculosis. They are taught to use handkerchief to cover their mouths when coughing or to wear face masks.” (p5, female, nurse)
  “Yes, it's part of counselling.”(p3, female, nurse)
  “Yes, it is highly important; after teaching, you ask them to tell you some of the points highlighted.” (p14, female, nurse)

#### 3.3.4. Methods of Supporting Treatment of the Patients

When the nurses were asked about the methods they use to support the treatment of their patients, different responses were given but more than one-third of the participants (8 of the 20) said they support the care of the patient by giving psychological support to both the patient and family members.

Some of their responses were as follows:

  “I try to provide psychological support for them because they would be on medication for a long time and I also tell them about the effects of noncompliance with medication regimen.” (p12, female, nurse)
  “I try to provide emotional support and reassurance.” (p7, female, nurse)

### 3.4. Theme 3: Strategies to Overcome Challenges

#### 3.4.1. Perception of Whether the Hospital Is Doing Enough to Prevent the Spread of TB

The nurses were asked if they think the hospital is doing enough to prevent the spread of TB and almost half of the participants (9 of the 20) said they thought the hospital is doing enough.

Some of their responses were as follows:

  “Yes, the hospital management is trying in terms of providing free drugs.” (p20, female, nurse)
  “Yes, I think the hospital is doing well but there's the need to improve in the provision of precaution materials.” (p3, female, nurse)
  “I do not think the hospital is trying enough because the issue of protective materials like good quality masks and gloves should not be overlooked; isolation ward is also essential.” (p13, female, nurse)

#### 3.4.2. Perception on If HIV and Tuberculosis Patients Should Be Separated on the Wards

The nurses were asked if they think HIV and TB patients should be separated on the wards and all the participants (all of the 20) said yes, HIV and TB patients should be separated on the wards.

Some of their responses were as follows:

  “A patient with HIV has a suppressed immune system and if HIV and tuberculosis patients are in the same ward, due to the immune suppression, the HIV patient might get tuberculosis. It's like nosocomial infection to that HIV patient.”
  “Yes, because a patient with tuberculosis is supposed to be nursed in an isolated ward.”
  “Well a patient with TB may also have HIV but where the patient has only HIV and because of the low immunity of HIV patients, they should be nursed separately” (p1, male, nurse)

#### 3.4.3. Policies That Protect Nurses from Exposure to TB at Work

The nurses were asked if there are any policies that protect nurses from exposure to TB at work and half of the participants (10 of the 20) said there are no policies that protect nurses.

Some of their responses were as follows:

  “I do not think there is any policy, whatever happens, happens.” (p20, female, nurse) “There are no policies that I know that protect nurses from exposure to tuberculosis at work here o.” (p9, female, nurse)

#### 3.4.4. Strategies to Overcome Nurses' Challenges

The nurses were asked about the strategies that could be used to overcome the challenges they face and multiple answers were given. Almost half (9 of the 20) of the participants said there should be a provision of adequate PPE.

Some of their responses were as follows:

  “We should have a policy on how to handle communicable diseases and also there should be provision of adequate personal protective equipment.” (p5, female, nurse)
  “The hospital should provide adequate necessities for care and should provide an isolation ward for better care and monitoring.” (p7, female, nurse)
  “There should be provision of good working environment, adequate provision of needed materials, provision of incentives, seminars, and training workshop to update our knowledge and skills in the care of TB cases.” (p15, male, nurse)

## 4. Discussion of Findings

The findings of this study denoted that all the participants expressed concern for their safety when caring for TB patients and their fear of infecting their immediate family members, relatives, and other patients. Reference [17] affirmed these findings where one of the challenges in caring for TB patients is the health workers' fear of getting infected with TB. Also [18] reported that participants expressed fear of contracting TB and carelessly infecting their immediate families, relatives, and others. The family members, therefore, constitute their interpersonal influences according to Pender's model [15] because the thought of infecting their family members with TB reduced the level of care given to the patients. Findings from this study showed that the fear of getting infected with TB affects the way some nurses care for the patients. Similarly, according to [19] in their study, only a few of the participants (resident doctors) reported empathy for TB patients and wished to help them while the remaining participants reported that though they have empathy, they avoid the patients for fear of being infected. Tudor, Van der Walt, and Farley also confirm this finding in their study in South Africa [20].

The study also sheds light on the challenges that nurses face in the care of TB patients. Some nurses talked about the inadequate PPE available in the hospital. Others talked about the lack of isolation wards for the patients to be nursed in. It is important to note here that almost half of the nurses that took part in this study work in the male medical ward and most of them talked about the lack of isolation ward for the proper care of TB patients because unlike the female ward where there is a small isolation ward for TB patient, the male ward did not have an isolation ward, hence the responses from the respondents. This finding was corroborated by Zelnick, Gibbs, Loveday, Padayatchi, and O‟Donnell [21], where the health workers also complained of the lack of isolation wards and lack of masks, and others talked about the length of time it takes to diagnose a tuberculosis patient. All these comments correlate with the findings in this study and that nurses still face challenges in caring for TB patients.

With regard to the roles of nurses in supporting the TB patients, it is important to denote that from the study carried out most of the nurses knew their roles in the care of TB patients. They give regular health talks to the patients concerning their compliance with their medication regimen and they teach them ways of preventing the spread of TB in their homes and communities.

The study also showed that nurses support the patients' treatment in various ways. Some nurses do support the patients by giving both the patients and the family members health talks, and some nurses give psychological support while others give follow-up care.

In the nurses' opinion regarding the strategies to overcome challenges, the majority of the participants said that the hospital is not doing enough to prevent the spread of infection pointing to the lack of isolation wards in the hospitals. Some nurses also talked about the fact that there are no adequate policies that protect nurses when caring for TB patients. The participants suggested such strategies as the provision of adequate PPE, so they can care for TB patients properly, provision of isolation wards for the patients to be nursed in, organization of seminars and training for nurses who handle TB patients, and provision of adequate staff.

The study established inadequate PPE, lack of isolation wards, delegating the care of TB patients to young inexperienced nurses, the length of time taken to diagnose a TB patient, lack of adequate policy that protects the care providers, and inadequate training as challenges and/or inadequacies that they face in TB care.

## 5. Conclusion

The major concern, as expressed by all the nurses, is the fear of getting infected with TB and inadvertently infecting their immediate families, relatives, and other patients. In addition, respondents expressed that senior nurses reassigning cases of TB patients to junior inexperienced officers also is a challenge. According to [22], some health workers highlighted the need for a refresher course and retraining on TB, prevention, and current treatment modalities stating that inadequate training is a challenge they face and this study supports this as some nurses identified a need to be trained adequately in managing TB patients.

### 5.1. Recommendations

*Hospital.* There should be provision of adequate protective equipment as well as provision of isolation wards for proper nursing of the patients.

*Government.* The State government should provide adequate policies that would protect nurses when caring for TB patients and also organize seminars periodically to update the nurses' skills so that they could care for TB patients adequately. Also, efforts should be intensified on an awareness campaign on how to curb the spread of TB and enlighten the members of the public on prevention/protection.

Fear and stigmatization among members of the communities, stakeholders, and health care workers may lead to absurd and discriminatory policy formulation with regard to TB management, so before policies are created, there should be information campaign about tuberculosis and its management at all tiers of the health care and in the community, using all available communication channels [23].

*Nurses.* Nurses should endeavour to take appropriate precautions when caring for these patients rather than neglecting them.

*Research.* Further studies should be conducted on the challenges that TB patients face in receiving treatment for TB as the finding(s) may guide the stakeholders in health planning and decision making. A quantitative study that actually quantifies the challenges to which nurses are exposed could strengthen the request for improved infrastructure and equipment.

### 5.2. Implication of the Study to Nursing

This study had shed light on some challenges that nurses face in the care of tuberculosis patients. The lack of adequate protective garments and an isolation ward for nursing the patients posed concerns to the nurses in charge of these patients.

The study recommended the availability of adequate PPE [24, 25], provision of isolation wards in all institutions managing TB patients, the separation of TB patients and HIV patients on the ward, and also having seminars, workshops, and retraining programmes periodically to update the nurses on current methods and techniques for protection against TB.

A standard, effective policy that should be adequately communicated to the nurses around the country should be drawn and adopted for the protection of the staff members and other patients against TB. The senior nurses should lead by example and thus mentor the young inexperienced professional colleagues. Nurses need to intensify efforts to educate the patients, their family members, and the community on ways of preventing the spread of TB so as to curb the spread of the disease.

### 5.3. Limitation of the Study

Because the study was conducted at one teaching hospital with purposive sampling involving only the nurses, the findings of the study cannot be generalized to a larger context in the health care industry. Future research needs to recruit nurses and other health care workers involving more hospitals.

## Figures and Tables

**Table 1:** Demographic data of participants (*n* = 20).

Demographic characteristics	Frequency	Percentage (%)
Sex		
Male	6	30.0
Female	14	70.0
Age		
21–30	2	10.0
31–40	11	55.0
41 and above	7	35.o
Marital status		
Single	3	15.0
Married	15	75.0
Separated	2	10
Ward		
Male medical ward	9	45.0
Female medical ward	5	25.0
Children's ward	6	30.0
Level/designation		
Chief Nursing Officer	3	15.0
Assistant Chief Nursing Officer	3	15.5
Principal Nursing Officer	1	5.0
Senior Nursing Officer	1	5.0
Nursing Officer I	2	10.0
Nursing Officer II	10	50.0
Length of time working with TB patients		
1–5 years	7	35.0
6–10 years	9	45.0
11–15 years	1	5.0
16 years and above	3	15.0

Twenty participants were interviewed (*n* = 20).

**Table 2:** Showing the themes and initial categories derived from the data.

Theme	Categories
Challenges of nurses	Concern for safety when working with patients
	Years working with patients
	Knowledge of MDR-TB
	Perception of MDR-TB
	Ways through which nurses might get exposed to TB at the workplace
	Activities that constitute TB exposure
	Measures for protection from TB at work
	Knowledge of whom the nurses fear might get infected if they contract TB
	Perceived effects of nurses' fear of the care of patients
	Perceived challenges of the nurses

Roles of nurses in supporting treatment	Giving health talks to patients concerning the effect of noncompliance with the medication regimen
	Follow-up and tracing of patients that fail to come for medications
	Teaching on ways of preventing the spread of TB in their homes and communities
	Methods of supporting treatment of the patients

Strategies to overcome challenges	Perception of whether the hospital is doing enough to prevent the spread of TB
	Perception of if HIV and TB patients should be separated on the wards
	Policies that protect nurses from exposure to TB at work
	Strategies to overcome nurses' challenges

## Data Availability

The data used to support the findings of this study are included within the article.

## References

[1] Berman A., Snyder S. (2012). *Kozier & Erb’s. Fundamentals of Nursing*.

[2] WHO (2017). *Global Tuberculosis Report*.

[3] Global Burden of Disease Collaborative Network (2017). *Global Burden of Disease Study 2016 (GBD 2016) Population Estimates 1950–2016*.

[4] Ahmad N. M. R., Montañola-Sales C., Prats C., Musa M., López D., Casanovas-Garcia J. (2018). Analyzing policymaking for tuberculosis control in Nigeria. *Complexity*.

[5] WHO (2010). *The Stop TB Strategy*.

[6] Uplekar M., Weil D., Lonnroth K. (2015). WHO’s new end TB strategy. *The Lancet*.

[7] World Health Organization (2018). *Multidrug and Extensively Drug-Resistant TB (M/XDRTB): 2010 Global Report on Surveillance and Response*.

[8] Karumbi J., Garner P. (2015). Directly observed therapy for treating tuberculosis. *Cochrane Database of Systematic Reviews*.

[9] Centers for Disease Control and Prevention (2018). Treatment for TB disease. https://www.cdc.gov/tb/topic/treatment/tbdisease.htm.

[10] Garfein R. S., Liu L., Cuevas-Mota J. (2018). Tuberculosis treatment monitoring by video directly observed therapy in 5 health districts, California, USA. *Emerging Infectious Diseases*.

[11] Centre for Disease Control and Prevention (2015). Fact sheet: trends in tuberculosis. http://www.cdc.gov/tb/publications/factsheets/statistics/tbtrend.htm.

[12] Sulis G., Centis R., Sotgiu G. (2016). Recent developments in the diagnosis and management of tuberculosis. *NPJ Primary Care Respiratory Medicine*.

[13] Dimitrova B., Balabanova D., Atun R., Drobniewski F., Levicheva V., Coker R. (2006). Health service providers’ perceptions of barriers to tuberculosis care in Russia. *Health Policy and Planning*.

[14] Makhado L., Davhana-Maselesele M., Farley J. E. (2018). Barriers to tuberculosis and human immunodeficiency virus treatment guidelines adherence among nurses initiating and managing anti-retroviral therapy in KwaZulu-Natal and North West provinces. *Curationis*.

[15] Pender N. J. (1996). *Health Promotion in Nursing Practice*.

[16] Iribaren S. J., Rubenstein F., Discacciati V., Pearce P. F. (2014). Listening to those at the frontline patient and health personnel perspective on tuberculosis treatment barriers and facilitators in high tuberculosis burden regions of Argentina. *Tuberculosis Research and Treatment*.

[17] Bulage L., Sekandi J., Kigenyi O., Mupere E. (2014). The quality of tuberculosis services in health care centres in a rural district in Uganda: the providers’ and clients’ perspective. *Tuberculosis Research and Treatment*.

[18] Brouwer M., Coelho E., Dores Mosse C. d., Brondi L., Winterton L. (2014). Healthcare workers’ challenges in the implementation of tuberculosis infection prevention and control measures in Mozambique. *PLoS One*.

[19] Geeta S. P., Dileep K., Ajay C. (2017). TB risk perceptions among medical residents at a tertiary care center in India. *Tuberculosis Research and Treatment*.

[20] Tudor C., Van der Walt M., Mphahlele M., Farley J. E. (2013). Health care workers’ fears associated with working in multidrug- and or extensively-resistant tuberculosis wards in South Africa. *The International Journal of Tuberculosis and Lung Disease*.

[21] Zelnick J. R., Gibbs A., Loveday M., Padayatchi N., O’Donnell M. R. N. (2013). Health-care workers’ perspectives on workplace safety, infection control, and drug-resistant tuberculosis in a high-burden HIV setting. *Journal of Public Health Policy*.

[22] Wynne A., Richter S., Banura L., Kipp W. (2014). Challenges in tuberculosis care in Western Uganda: health care workers and patients’ perspectives. *International Journal of African Nursing Sciences*.

[23] van Cutsem G., Isaakidis P., Farley J., Nardell E., Volchenkov G., Cox H. (2016). Infection control for drug-resistant tuberculosis: early diagnosis and treatment is the key: table 1. *Clinical Infectious Diseases: An Official Publication of the Infectious Diseases Society of America*.

[24] World Health Organization (2012). *Essential Biosafety Measures for TB Laboratories*.

[25] Federal Ministry of Health/WHO (2008). *The National Guidelines for TB Infection Control*.

